# Cancer Risk in Thyroid Nodules: An Analysis of Over 1000 Consecutive FNA Biopsies Performed in a Single Canadian Institution

**DOI:** 10.3390/diagnostics14242775

**Published:** 2024-12-10

**Authors:** Elsabe J. Smit, Sana Samadi, Mitchell P. Wilson, Gavin Low

**Affiliations:** Department of Radiology and Diagnostic Imaging, University of Alberta Hospital, Edmonton, AB T6G2B7, Canada; elsabe@ualberta.ca (E.J.S.); ssamadi2@ualberta.ca (S.S.); mitch.wilson@ualberta.ca (M.P.W.)

**Keywords:** thyroid, thyroid nodule, thyroid cancer, ultrasound, TI-RADS, ACR TI-RADS, risk of malignancy, fine needle aspiration (FNA)

## Abstract

**Objective:** To determine the cancer risk in thyroid nodules using ACR TI-RADS. **Methods:** A retrospective analysis of all thyroid biopsies was performed over a 3-year period (2021 to 2023). Variables including gender, age, history of thyroid cancer or neck irradiation, nodule size and location, TR level, and sonographic features such as punctate echogenic foci (PEF), a very hypoechoic appearance, taller-than-wide shape, and suspected extrathyroidal extension were analyzed. **Results:** A total of 1140 nodules were assessed in 993 patients, including 740 females (74.5%) and 253 males (25.5%). The mean patient age was 57.1 ± 15.4 years. Variables significantly associated with nodule malignancy included (1) younger age, (2) a prior history of thyroid cancer or neck irradiation, (3) a higher TR level, (4) a taller-than-wide shape in nodules <1 cm, (5) PEF, (6) a very hypoechoic appearance, and (5) suspected extrathyroidal extension (*p* < 0.05). Gender, nodule location and size were not associated with a higher cancer risk (*p* > 0.05). Malignancy was found in 40.7% of TR5, 4.8% of TR4, 0.3% of TR3, and 0% of TR1 and 2 nodules. The odds ratios (ORs) for cancer were as follows: TR4 or 5, OR = 19; PEF, OR = 11; a very hypoechoic appearance, OR = 13.3; and suspected extrathyroidal extension, OR = 27.2 (*p* < 0.01). **Conclusions:** Higher TR levels, PEF, a very hypoechoic appearance, and suspected extrathyroidal extension are important features for predicting cancer risk. These findings affirm the effectiveness of ACR TI-RADS in nodule risk stratification.

## 1. Introduction

Nodular thyroid disease is very common in the general population [1]. Its prevalence increases with age and is found in 30 to 40% of persons over 50 [2,3,4]. It has a strong female predilection, with a female-to-male ratio of 4 to 1 [2,3,4,5]. The majority of nodules are clinically silent and are incidentally discovered on imaging [1,3]. These are found in up to 68% on ultrasound (US), in 15% on computed tomography (CT) or magnetic resonance imaging (MRI), and in 1–2% on positron emission tomography (PET) [6,7,8,9,10,11]. The increased incidence of thyroid nodules compared to that in previous decades has been attributed to escalating imaging volumes and improvements in modern imaging techniques, which are more accurate in detecting small nodules [12,13,14,15]. However, most thyroid nodules are benign, with malignancy observed in around 10% [16,17,18,19,20,21]. Most thyroid malignancies, particularly papillary cancer, which accounts for 80–85%, are slow-growing and have indolent behaviour. They do not generally lead to death when cancers are small and localized or in the elderly, where mortality occurs due to other comorbidities. The increased detection of thyroid nodules has led to a surge in referrals for US-guided fine needle aspiration (FNA) biopsies and for surgical intervention, mostly in small papillary cancers. Despite this, the overall mortality rate from thyroid cancer has remained unchanged, implying that most operations do not confer a survival benefit [5,6,12,22,23]. This epidemic of overdiagnosis and overtreatment of thyroid nodules represents a socioeconomic burden with implications for healthcare systems worldwide [5,22]. It may also cause physical and psychological harm due to patient anxiety, loss of workdays, and post-operative morbidity. In the United States alone, between 2004 and 2007, it is estimated that overdiagnosis of thyroid cancer was responsible for 70–80% of cases in females and 45% in males [6,13].

To address these problems, various US-based classification systems have been developed to risk-stratify thyroid nodules with the intention that patients with high-risk nodules may be referred for biopsy, patients with intermediate-risk nodules may be managed using imaging follow-up, while those with low-risk nodules may be reassured. Additionally, these systems have developed their own ultrasound lexicon to improve thyroid nodule characterization and reduce the subjectivity of the imaging interpretation. This has the advantage of promoting consistency in radiology reports, including standardized recommendations based on risk categories; reducing reporting ambiguity through uniform terminology; and encouraging a more coherent flow of information to referring physicians, thus optimizing interdisciplinary communication [6,24,25,26]. Some of the most well-known classification systems include the American College of Radiology (ACR) TI-RADS (Thyroid Imaging, Reporting, and Data System), the European Thyroid Imaging and Reporting Data System (EU-TIRADS), and the Korean Thyroid Imaging Reporting and Data System (K-TIRADS) [1,6,15,27,28,29]. As ACR TI-RADS is the most widely used system worldwide, it is held up as the primary focus of evaluation in this article. Briefly, nodule evaluation in ACR-TIRADS is based on five morphological features on ultrasound: ***‘composition’***, ***‘echogenicity’***, ***‘shape’***, ***‘margins’***, and ***‘echogenic foci’***. For each feature, a point score is assigned, with low-risk features assigned ‘+0 or +1 points’, intermediate-risk features ‘+2 points’, and high-risk features ‘+3 points’. The ‘+3-point’ high-risk features include a ‘hypoechoic appearance,’ a ‘taller-than-wide’ shape, ‘extrathyroidal extension,’ and ‘punctate echogenic foci’ [6,15]. The points across all five morphological features are summed to give a total point score. This score is used to risk-stratify nodules into the following TI-RADS categories: **TR1** *‘benign’* (0 points), **TR2** *‘not suspicious’* (2 points), **TR3** *‘mildly suspicious’* (3 points), **TR4** *‘moderately suspicious’* (4–6 points), and **TR5** *‘highly suspicious’* (≥ 7 points). The TR level is then combined with the maximum nodule size to produce a set of recommendations, as described in Table 1.

Li et al. performed a meta-analysis and a systematic review of the diagnostic performance of ACR TI-RADS in 16 studies involving 18,614 patients and 21,882 nodules [30]. The authors found that ACR TI-RADS had a pooled sensitivity of 0.89 (95% CI, 0.81 to 0.93), a pooled specificity of 0.7 (95% CI, 0.6 to 0.78), an area under the receiver operating curve (AUROC) of 0.86 (95% CI, 0.83 to 0.89), and an odds ratio (OR) of 18.46 (95% CI, 9.77 to 34.88). The authors concluded that ***‘the use of ACR TI-RADS could avoid a large number of unnecessary biopsies, although at the cost of a slight decline in sensitivity’*** [30]. A meta-analysis and a systematic review by Kim et al., involving 29 articles and 33,748 nodules, found pooled sensitivities and specificities for malignant nodules of 66% (95% CI, 56 to 75%) and 91% (95% CI, 87 to 94%) for ACR TI-RADS, 82% (95% CI, 71 to 89%) and 90% (95% CI, 77 to 96%) for EU-TIRADS, and 55% (95% CI, 38 to 70%) and 95% (95% CI, 90–98%) for K-TIRADS [31]. The authors concluded that ***‘the overall diagnostic performance of the US-based classification systems were comparable’*** [31]. In a meta-analysis and a systematic review by Kim et al. involving eight articles and 13,092 nodules, ACR TI-RADS was found to have the lowest unnecessary biopsy rate, at 25% (95% CI, 22 to 29%), compared with 38% (95% CI, 16 to 66%) for EU-TIRADS and 55% (95% CI, 42 to 67%) for K-TIRADS [23].

Our institution in Edmonton, Alberta, Canada, serves as the largest teaching hospital in the city and a referral center for ultrasound-guided FNA biopsy of thyroid nodules. Our metropolitan catchment area encompasses approximately 1.6 million people. This study aimed to determine the cancer risk in thyroid nodules using ACR TI-RADS within our population and to correlate our findings with the existing medical literature. We reviewed all thyroid nodules that underwent US-guided biopsy during a three-year consecutive period to ensure a comprehensive evaluation of our findings.

## 2. Materials and Methods

### 2.1. Ethics and Institutional Approval

This study was conducted in accordance with the Declaration of Helsinki, and the protocol was approved by the Ethics Committee of the University of Alberta (HREBA.CC-24-0099) on 4 April 2024. This study received a waiver of written patient consent, as all cases were anonymized, and personal identifying information was removed.

### 2.2. Study Design

A retrospective observational study was performed at the University of Alberta Hospital. The purpose of this study was to determine the cancer risk in thyroid nodules based on the following:

- Gender;
- Age;
- Prior history of thyroid cancer or neck irradiation;
- Nodule location;
- Nodule size;
- TR level;
- High-risk US features, including punctate echogenic foci, taller-than-wide shape, a very hypoechoic appearance, and suspected extrathyroidal extension.

### 2.3. Study Population

The study population included all consecutive patients who underwent an US-guided FNA thyroid biopsy over a three-year period between 1 January 2021 and 31 December 2023. There were no study exclusions.

### 2.4. US-Guided Fine Needle Aspiration (FNA) Biopsy

Most of our referrals were from family medicine, based on reported findings from thyroid ultrasound examinations conducted at various outpatient radiology clinics in our city.

At our institution, all US-guided thyroid biopsies were performed by board-certified staff radiologists using a high-frequency linear array ultrasound transducer (6–15 MHz) on a LOGIQ E9 platform (GE HealthCare, Chicago, IL, USA). 22-gauge needles were used for FNA. The cytopathology results of the FNA biopsies were reported using the Bethesda system (2017).

### 2.5. Data Collection

The picture archiving and communication system (PACS) and provincial electronic health records were accessed to review the thyroid US images, radiology reports, and pathology results. A fellowship-trained board-certified staff radiologist with 17 years of experience reviewed the findings. The extracted data were anonymized and entered into a password-protected Excel spreadsheet for analysis.

### 2.6. AI Tools

AI tools were used only to identify and correct grammatical and spelling errors.

### 2.7. Statistical Analysis

Categorical variables were expressed as values and percentages. Continuous variables were expressed as the mean ± standard deviation. Statistical analysis was performed on IBM SPSS (version 29). A *p* value of <0.05 was considered statistically significant. The following statistical tests were performed:

- Chi-squared test;
- Independent samples *t*-test;
- Binomial logistic regression;
- Receiver operating characteristic (ROC) analysis.

## 3. Results

### 3.1. Clinical Information

There were 993 patients, including 740 females (74.5%) and 253 males (25.5%). No significant differences were observed in the prevalence of malignant thyroid nodules based on gender (*p* = 0.56). Malignant nodules were found in 9.8% of males compared with 8.5% of females. The patients’ mean age was 57.1 ± 15.3 years and ranged from 15 to 93 years. The average age of patients with malignant nodules (48.4 ± 16.5 years) was not significantly different from those with benign nodules (57.6 ± 16.5 years) (*p* = 0.18). However, when the data were analyzed by age group (<30 years, 30 to 60 years, and >60 years), a significant association was observed between younger age cohorts and a higher frequency of cancer (*p* < 0.001). Malignant nodules were found in 18.4% of patients under 30, compared to 11.9% in those aged between 30 and 60, and in 4.5% of individuals over 60. A past medical history of thyroid cancer or neck irradiation was observed in 15 (1.5%). A significantly higher frequency of malignant nodules was found in patients with (27.3%) a past history of cancer or neck irradiation compared to those without (8.2%) this history (*p* = 0.02).

### 3.2. Nodule Pathology

There were 1140 nodules in the 993 patients, of which 1014 nodules in 884 patients were either benign or malignant. The findings on FNA are included in Table 2.

Of the 85 malignant nodules, papillary cancer was observed in 78 (91.8%), medullary cancer in 1 (1.2%), anaplastic cancer in 1 (1.2%), lymphoma in 1 (1.2%), and lung cancer metastases in 2 (2.4%).

### 3.3. Nodule Location

Table 3 includes the location of benign and malignant nodules in the thyroid gland. Nodule location was not associated with an increased risk of cancer (*p* = 0.08).

### 3.4. Nodule Size

The mean nodule size was 2.6 ± 1.3 cm and ranged from 0.5 to 9.8 cm. There were 23 nodules (2.3%) <1 cm and 991 (97.7%) ≥1 cm. The mean size of the benign nodules (*n* = 929) was 2.6 ± 1.3 cm. The mean size of the malignant nodules (*n* = 85) was 2.2 ± 1.4 cm. There were no significant differences in the mean nodule size between the benign and malignant nodules (*p* = 0.53).

### 3.5. TR Level

The TR level of the nodules is included in Table 4.

The prevalence of benign and malignant nodules per TR level is included in Table 5. Higher TR levels were associated with a significantly greater risk of cancer (*p* < 0.001).

### 3.6. Presence of High-Risk US Features

A. *Punctate echogenic foci (PEF)*

The prevalence of PEF is included in Table 6. PEF were associated with a significantly greater risk of cancer (*p* < 0.001).

B. *Taller-than-wide*

The prevalence of a taller-than-wide shape is summarized in Table 7. Overall, a taller-than-wide shape was not associated with an increased risk of cancer (*p* = 0.40) when nodules of all sizes were evaluated collectively. However, a subgroup analysis revealed that for nodules <1 cm, a taller-than-wide shape was significantly more common in malignant nodules compared to benign nodules (*p* = 0.03). Of the 23 nodules (20 benign and 3 malignant) that were <1 cm, a taller-than-wide shape was seen in 66.7% of the malignant nodules compared to only 5% of the benign nodules. Interestingly, for nodules ≥1 cm, the subgroup analysis showed no significant difference in the frequency of a taller-than-wide shape between malignant (14.6%) and benign nodules (13%) (*p* = 0.67).

C. *Very hypoechoic appearance*

The prevalence of a very hypoechoic appearance is included in Table 8**.** A very hypoechoic appearance was associated with a significantly greater risk of cancer (*p* < 0.001).

D. *Suspected extracapsular extension*

The prevalence of suspected extracapsular extension is included in Table 9. Suspected extracapsular extension was associated with a significantly greater risk of cancer (*p* < 0.001).

E. *Multiple high-risk US features*

The prevalence of multiple high-risk US features is included in Table 10. The presence of multiple high-risk US features was associated with a significantly greater risk of cancer (*p* < 0.001).

### 3.7. Regression Model

Binomial logistic regression was performed to determine the effects of TR level and high-risk US features such as PEF, a very hypoechoic appearance, and suspected extrathyroidal extension on the cancer risk in thyroid nodules. Taller-than-wide shape was not included in the model, as this was not significant for cancer prediction when nodules of all sizes were evaluated collectively. The regression model was significant, with χ^2^(4) = 193 and *p* < 0.001. The model explained 40% (Nagelkerke R^2^) of the variance in cancer and correctly classified 92.6% of cases. Its sensitivity was 14.1%, its specificity was 99.8%, its positive predictive value was 85.7%, and its negative predictive value was 92.7%. In the multivariate analysis, the ORs for cancer were as follows:

- TR 4 or 5: OR = 19 (95% CI, 2.6 to 140.9), *p* = 0.004
- PEF: OR = 11 (95% CI, 6.4 to 18.8), *p* < 0.001
- Very hypoechoic appearance: OR = 13.3 (95% CI, 3.8 to 45.9), *p* < 0.001
- Suspected extrathyroidal extension: OR = 27.2 (95% CI, 4.6 to 160), *p* < 0.001

**Figure 1:**
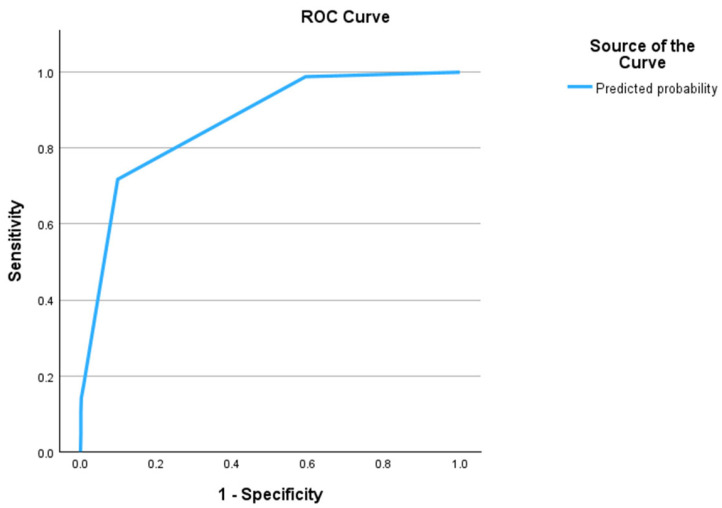
Predictive value of the regression model for nodule malignancy.

The ROC analysis (Figure 1) of the regression model showed an AUROC of 0.87 (95% CI, 0.83 to 0.91).

## 4. Discussion

In line with the medical literature, we found a strong female preponderance for the prevalence of thyroid nodules, with a female-to-male ratio of 3:1 [2,3,4,5]. Despite this, our findings showed no significant gender differences in the frequency of malignant nodules, with rates of 9.8% in males vs. 8.5% in females (*p* = 0.56). A meta-analysis by LeClair et al. proposed that the perceived gender disparities in thyroid cancer may be due to higher detection rates in females, attributed to differences in healthcare utilization patterns rather than underlying biological differences [32]. The authors noted that the pooled post mortem prevalence of subclinical papillary thyroid cancer was comparable, with rates of 11% (95% CI, 5 to 18%) in males and 14% (95% CI, 8 to 20%) in females [32].

We found a significant association between younger age groups and a higher frequency of thyroid cancer (*p* < 0.001). Malignant nodules were observed in 18.4% of patients under 30 years old compared to 11.9% in those aged between 30 and 60, and 4.5% in patients over 60. The mechanisms contributing to the higher cancer frequency among younger cohorts are unclear. A study by Belfiore found that malignant nodules were more common in patients under 30 or over 60 [33]. In a study by Kwong et al. involving 6654 patients and 12,115 nodules, the prevalence of thyroid nodules was reported to increase with age from 1.5 in the 20- to 30-year-old age group to 2.2 in those over 70 (*p* < 0.01) [34]. However, when analyzed on a per nodule basis, the cancer risk was highest in patients aged between 20 and 30, with malignancy found in 14.8% of this age group, compared to 5.6% of individuals over 70 (*p* < 0.01) [34]. The authors found that each additional year between the ages of 20 and 60 was associated with a 2.2% relative reduction in the risk of a newly evaluated thyroid nodule being malignant (OR = 0.97, *p* < 0.001) [34].

In our study, a prior history of thyroid cancer or neck irradiation was significantly associated with a higher rate of malignancy in newly evaluated thyroid nodules (*p* = 0.02). Within this cohort, 27.3% of nodules were malignant, compared to only 8.2% in patients without this history. Our findings agree with the medical literature [4,35]. Iglesias et al. noted that the main risk factors for thyroid cancer following radiation exposure include (i) the radiation dose and (ii) the age at which the exposure occurred, with an increased risk for doses exceeding 0.05 to 0.1 Gy and for childhood exposures [35]. The authors reported a minimum latency period of 5 to 10 years to cancer onset, with papillary cancer being the most common type [35].

The nodule location within the thyroid gland and its usefulness in predicting cancer have been the subject of recent interest [36,37,38,39]. In our study, 48% of nodules were located in the right lobe, 45.3% in the left lobe, and 6.8% in the isthmus. The prevalence of malignancy was highest in the isthmus at 12.1% compared to 9.8% in the right lobe and 6.3% in the left lobe. However, these findings were not significant (*p* = 0.08). In a study of 3241 thyroid nodules, of which 10.3% were malignant, Jasim et al. found that nodule location was an independent risk factor for cancer, with isthmic nodules—despite them being the least common, at 6%—having the highest risk (OR = 2.4, 95% CI 1.6 to 3.6) (*p* = 0.005) [36]. Pastorello et al., in a study including 9535 FNA biopsies, found a significantly higher prevalence of malignancy in isthmic nodules (8.1%) compared to nodules in the right (3.6%) or left (3.1%) lobes (*p* < 0.001) [39]. At present, there is no clear explanation for why isthmic nodules may have a higher cancer risk. Nevertheless, reports suggest that isthmic cancers have a worse prognosis compared to those found elsewhere due to the propensity for (i) extrathyroidal extension and (ii) lymph node metastases [39,40,41].

There are conflicting reports regarding the association between nodule size and cancer risk [42,43,44]. Our study showed no significant difference in the mean size of benign and malignant nodules (2.6 ± 1.3 cm vs. 2.2 ± 1.4 cm) (*p* = 0.53). A meta-analysis by Hammad et al., which included seven studies with a total of 10,817 nodules, found that nodules between 3 and 5.9 cm had a 26% higher cancer risk compared to nodules <3 cm (OR 1.26, 95% CI, 1.13 to 1.39) [43]. Additionally, the authors noted that nodules ≥6 cm had a 16% lower cancer risk compared to nodules <3 cm (OR 0.84, 95% CI, 0.73 to 0.98) [43]. Shayganfar et al. observed an inverse relationship between cancer risk and nodule size, with a cut-off of <12 mm being deemed suspicious [42]. Scorziello et al. performed an analysis involving TR3 to 5 nodules and found that size was not a significant predictor of cancer within these TR levels [44]. Where size becomes important is with respect to the prognosis in malignant nodules, with larger nodules being associated with worse outcomes due to a higher risk for local invasion and metastatic disease [45,46].

Our findings support the effectiveness of ACR TI-RADS in classifying nodules into TR levels 1 to 5 on the basis of cancer risk. Within our study population, the cancer rates were 0% for TR1 and 2 *(‘benign’*), 0.3% for TR3 (*‘mildly suspicious’*), 4.8% for TR4 (*‘moderately suspicious’*), and 40.7% for TR5 (*‘highly suspicious’*). In a multicenter study conducted in the United States involving 3422 nodules, of which 352 were malignant, Middleton et al. reported cancer rates of 0.3% for TR1, 1.5% for TR2, 4.8% for TR3, 9.1% for TR4, and 35% for TR5 [45]. In a multicenter study conducted in China involving 2465 nodules, of which 1005 were malignant, Xu et al. reported cancer rates of 0% for TR1, 2.3% for TR2, 7.5% for TR3, 40.1% for TR4, and 81.4% for TR5 [47]. These studies indicate that higher TR levels are associated with a higher cancer risk.

**Figure 2:**
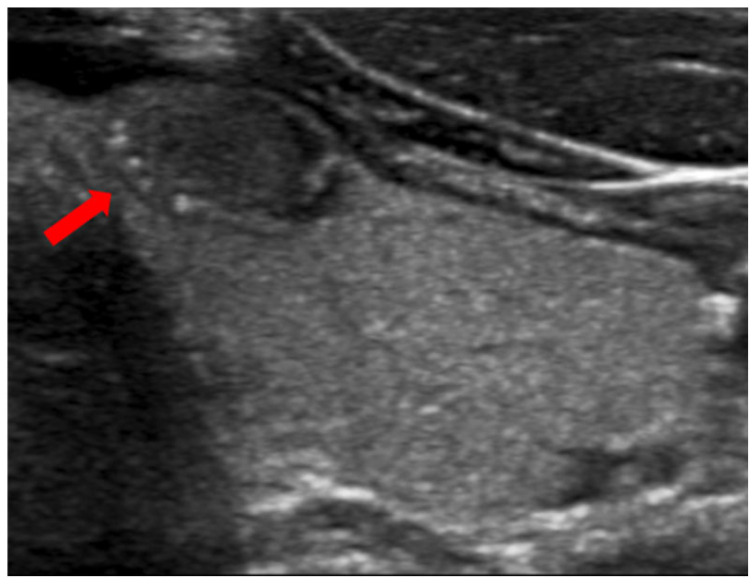
A malignant nodule (arrow) with punctate echogenic foci.

PEF refers to distinct echogenic dots ≤1 mm within a thyroid nodule (Figure 2) [24]. Unlike macrocalcifications, PEF do not cast a posterior acoustic shadow, although they can be associated with small comet tail artifacts. It is important to differentiate PEF from echogenic foci > 1 mm, which can be associated with more pronounced V-shaped comet tail artifacts; the latter are characteristic findings in benign colloid nodules [24]. The underlying etiology in PEF is unclear; in some instances, these may represent psammoma bodies in papillary cancers, while in others, they may indicate the back wall of microcysts [24]. PEF were observed in 64.7% of the malignant nodules compared to only 9% of the benign nodules in our study. Multivariate regression revealed that PEF were associated with a significant cancer risk, yielding an OR of 11 (95% CI, 6.4 to 18.8), at *p* < 0.001. Our findings agree with the literature. Nabahati et al. conducted a study involving 1129 nodules and found a significant correlation between PEF and malignancy, with ORs of 3.7 (95% CI, 2.3 to 5.8) in a univariate analysis and 1.9 (95% CI, 1.1 to 3.2) in a multivariate analysis [48]. Sohn et al. examined 1018 nodules and found a significant correlation between intra-solid PEF without comet tail artifacts and cancer risk, yielding an OR = 8.1 (95% CI, 3.6 to 17.9), at *p* < 0.001 [49]. Lastly, Ha et al. studied 1112 nodules and reported that PEF with comet tail artifacts were associated with a malignancy rate of 77.8% in solid and predominantly solid nodules [50].

**Figure 3:**
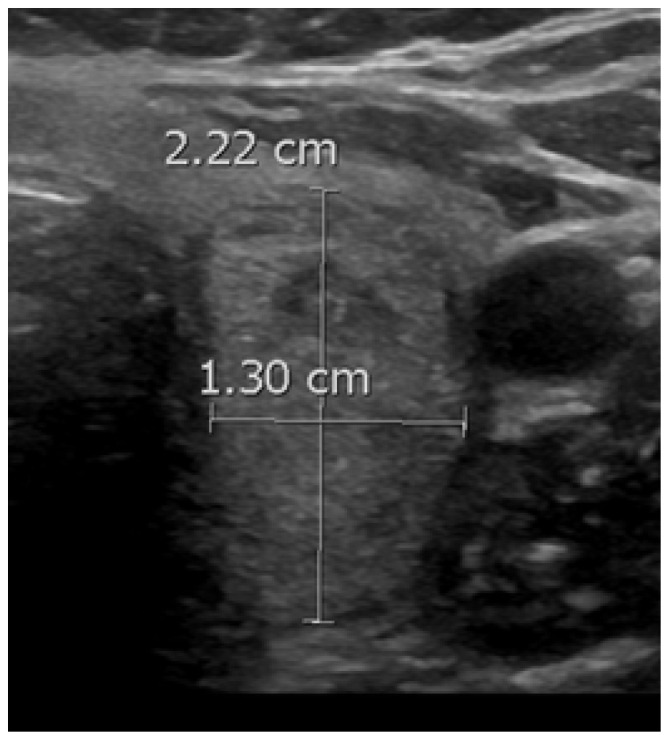
A benign nodule with a taller-than-wide shape.

When a thyroid nodule has an anteroposterior (AP) dimension that exceeds its transverse (TR) dimension such that the ratio is greater than 1, it is classified as having a taller-than-wide shape (Figure 3). This observation was first reported in 2002 by Kim et al. in a study involving 155 thyroid nodules with an average size of 7.5 mm, of which 49 were malignant. This study found that a taller-than-wide shape was associated with a 92.5% specificity and 32.7% sensitivity for malignancy [51]. The authors proposed that this taller-than-wide configuration may reflect an underlying tendency for malignant nodules to grow across tissue planes, unlike benign nodules, that grow along tissue planes [51]. A meta-analysis and a systematic review by Brito et al., examining 31 studies and 18,288 nodules with an average size of 1.5 cm, reported that a taller-than-wide shape was associated with an OR of 11.1 (95% CI, 6.6 to 18.9) for malignancy [52]. In our study, we observed a significant association between a taller-than-wide shape and malignancy for nodules <1 cm but not for those above this size. For nodules <1 cm, a taller-than-wide shape was observed in 66.7% of malignant nodules compared to 5% of benign nodules (*p* = 0.03). In comparison, for nodules ≥1 cm, a taller-than-wide shape was seen in 14.6% of malignant nodules versus 13% of benign nodules (*p* = 0.67). Other studies have found similar findings [53,54]. In a study involving 207 nodules, of which 110 were malignant, Ren et al. found that a taller-than-wide shape was a good predictor for papillary cancer for nodules ≤1 cm but not for larger nodules [53]. In a study involving 1238 nodules, of which 159 were papillary cancers, Kim et al. found that a taller-than-wide shape was associated with an OR for cancer of 2.7 (95% CI, 1.7 to 4.3) (*p* < 0.001) for nodules ≤1 cm vs. an OR of 1.2 (95% CI, 0.4 to 3.2) (*p* = 0.77) for nodules >1 cm [54].

**Figure 4:**
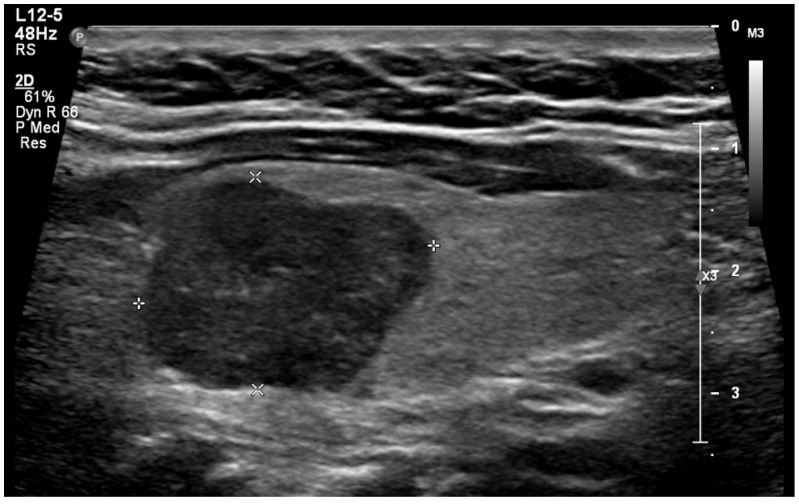
A malignant nodule with a very hypoechoic appearance.

Thyroid nodules that exhibit lower echogenicity than the adjacent neck muscles are described as having a very hypoechoic or markedly hypoechoic appearance (Figure 4) [6,15,24]. Our study found a very hypoechoic appearance in 9.4% of malignant nodules compared to 0.6% of benign nodules. This characteristic was significantly associated with malignancy, yielding an OR of 13.3 (95% CI, 3.8 to 45.9), at *p* < 0.001. In a study involving 2255 nodules, of which 293 were malignant, Lee et al. discovered that for homogenously solid nodules, a marked hypoechoic or moderate hypoechoic appearance was associated with a significantly higher cancer risk (51.9 to 59.2% vs. 21.3%) compared to a mildly hypoechoic appearance (*p* < 0.001) [55]. In a multi-institution study involving 5601 nodules, of which 1089 were malignant, Lee et al. observed a malignancy risk of 64.8% in markedly hypoechoic nodules, 48.2% in moderately hypoechoic nodules, 23.5% in mildly hypoechoic nodules, and 8.7% in iso- or hyperechoic nodules (*p* < 0.001) [56]. Kim et al. examined the relationship between the histologic findings and nodule echogenicity, observing a trend of increasing fibrosis as the nodule echogenicity decreased [57]. These study findings may help explain the tendency for markedly hypoechoic nodules to be malignant, as fibrosis is an underlying feature in papillary cancer [57].

**Figure 5:**
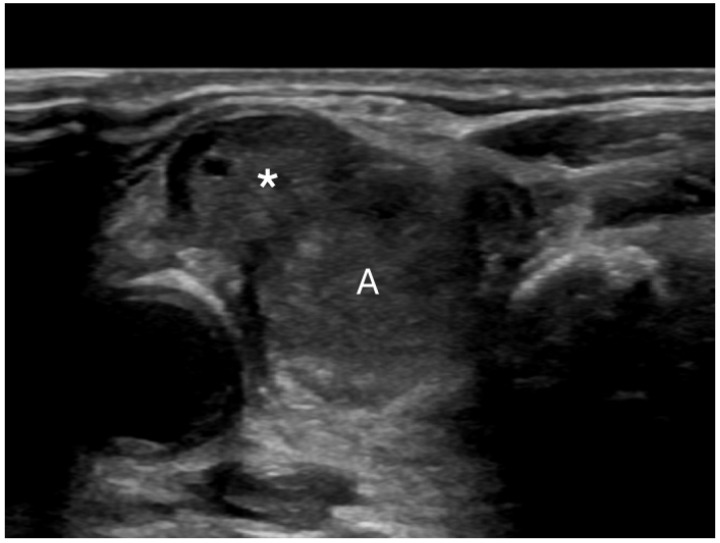
Extrathyroidal extension (*) extending anteriorly from a malignant nodule (A).

Extrathyroidal extension beyond the thyroid capsule into the surrounding soft tissues is reported in 5% to 34% of thyroid cancers and is a predictor of negative outcomes (Figure 5) [58,59,60]. Detecting minimal extrathyroidal extension, indicated by subtle capsular disruption, is challenging. Additionally, ill-defined benign nodules located near the thyroid capsule may cause focal bulging and capsular distortion, leading to false positives. Consequently, there is considerable interobserver variability in detecting extrathyroidal extension [61]. Lee et al. found that the combination of tumor disruption of the capsule—manifesting as a loss of the capsule’s outline—and >50% tumor contact with the capsule had 95.4% specificity, 23.7% sensitivity, and an AUROC of 0.64 for predicting extrathyroidal extension [25,62]. Our study found that 0.2% of benign nodules were misclassified as showing extrathyroidal extension. In contrast, extrathyroidal extension was found in 8.2% of malignant nodules. Overall, suspected extrathyroidal extension was associated with an OR for cancer of 27.2 (95% CI, 4.6 to 160), at *p* < 0.001.

This study had several limitations. The retrospective study design restricted nodule assessment to the sonographic images available on PACS. There was substantial heterogeneity in the referral practices in our city that may have impacted our study population. While the ACR TI-RADS recommendations for biopsy were adopted as guidelines, these were not enforceable for medical–legal reasons. Finally, the reference standard was FNA rather than surgical resection, which may have resulted in a small number of false negatives.

## 5. Conclusions

Our work has shown that cancer risk in thyroid nodules is associated with higher TR levels and with sonographic features such as PEF, a very hypoechoic appearance, and suspected extrathyroidal extension. Additionally, a higher cancer risk was observed in individuals between 20 and 30 years of age, in patients with a prior history of thyroid cancer or neck irradiation, and for taller-than-wide nodules <1 cm. Cancer risk was not influenced by gender, nodule location, or nodule size.

## Figures and Tables

**Table 1:** ACR TI-RADS recommendations.

Level	Follow-Up	FNA
**TR1**	no	no
**TR2**	no	no
**TR3**	at 1, 3, and 5 years if ≥1.5 cm and <2.5 cm	≥2.5 cm
**TR4**	at 1, 2, 3, and 5 years if ≥1 cm and <1.5 cm	≥1.5 cm
**TR5**	every year up to 5 years if ≥0.5 cm and <1 cm	≥1 cm

**Table 2:** Cytological findings on FNA.

Cytological Findings	Frequency	Percentage (%)
Benign	929	81.5
Malignant	85	7.5
Follicular neoplasm or suspicious for follicular neoplasm	20	1.8
Undetermined significance	77	6.8
Inadequate biopsy	29	2.5
Total	1140	100

**Table 3:** Nodule location.

Location	Benign	Malignant	Total
Right	443 (90.2%)	48 (9.8%)	491(100%)
Left	428 (93.7%)	29 (6.3%)	457 (100%)
Isthmus	58 (87.9%)	8 (12.1%)	66 (100%)

**Table 4:** TR level of nodules.

TR Level	Frequency	Percentage (%)
1	7	0.6
2	72	6.3
3	340	29.8
4	562	49.3
5	159	13.9
Total	1140	100

**Table 5:** The prevalence of benign and malignant nodules by TR level.

TR Level	Benign	Malignant
1	5 (100%)	0 (0%)
2	62 (100%)	0 (0%)
3	312 (99.7%)	1 (0.3%)
4	461 (95.2%)	23 (4.8%)
5	89 (59.3%)	61 (40.7%)
Total	929 (91.6%)	85 (8.4%)

**Table 6:** Punctate echogenic foci (PEF).

Punctate Echogenic Foci	Benign	Malignant
Present	84 (9%)	55 (64.7%)
Absent	845 (91%)	30 (35.3%)
Total	929 (100%)	85 (100%)

**Table 7:** Taller-than-wide shape.

Taller-Than-Wide	Benign	Malignant
**Nodules of All Sizes**		
Present	119 (12.8%)	14 (16.5%)
Absent	810 (87.2%)	71 (83.5%)
Total	929 (100%)	85 (100%)
**Nodules <1 cm**		
Present	1 (5%)	2 (66.7%)
Absent	19 (95%)	1 (33.3%)
Total	20 (100%)	3 (100%)
**Nodules ≥1 cm**		
Present	118 (13%)	12 (14.6%)
Absent	791 (87%)	70 (85.4%)
Total	909 (100%)	82 (100%)

**Table 8:** Very hypoechoic appearance.

Very Hypoechoic Appearance	Benign	Malignant
Present	6 (0.6%)	8 (9.4%)
Absent	923 (99.4%)	77 (90.6%)
Total	929 (100%)	85 (100%)

**Table 9:** Suspected extrathyroidal extension.

Suspected Extrathyroidal Extension	Benign	Malignant
Present	2 (0.2%)	7 (8.2%)
Absent	927 (99.8%)	78 (91.8%)
Total	929 (100%)	85 (100%)

**Table 10:** Multiple high-risk features.

Multiple High-Risk Features	Benign	Malignant
Present	10 (1.1%)	18 (21.2%)
Absent	919 (98.9%)	67 (78.8%)
Total	929 (100%)	85 (100%)

## Data Availability

The original contributions presented in this study are included in the article; further inquiries can be directed to the corresponding author.
